# Association between quality of life and various aspects of intradialytic hypotension including patient-reported intradialytic symptom score

**DOI:** 10.1186/s12882-019-1366-2

**Published:** 2019-05-14

**Authors:** Johanna Kuipers, Jurjen K. Oosterhuis, Wolter Paans, Wim P. Krijnen, Carlo A. J. M. Gaillard, Ralf Westerhuis, Casper F. M. Franssen

**Affiliations:** 1 Dialysis Center Groningen, Hanzeplein 1, 9713 GZ Groningen, The Netherlands; 2 Department of Anesthesiology, University Medical Center Groningen, University of Groningen, Groningen, The Netherlands; 3 Hanze University Groningen, University of Applied Sciences, Groningen, The Netherlands; 4 University of Utrecht Medical Center, University of Utrecht, Utrecht, The Netherlands

**Keywords:** Haemodialysis, Intradialytic hypotension, Quality of life, Patient reported outcome measures

## Abstract

**Background:**

There is increasing awareness that, besides patient survival, Quality of Life (QOL) is a relevant outcome factor for patients who have a chronic disease. In haemodialysis (HD) patients, intradialytic hypotension (IDH) is considered one of the most frequent complications, and this is often accompanied by symptoms. Several studies have investigated QOL in dialysis patients, however, research on the association between intradialytic symptoms and QOL is minimal. The goal of this study was to determine whether the occurrence of IDH has an influence on the perception of QOL.

**Methods:**

During 3 months, haemodynamic data, clinical events, and interventions of 2623 HD-sessions from 82 patients were prospectively collected. The patients filled out a patient-reported intradialytic symptom score (PRISS) after each HD session. IDH was defined according to the EBPG as a decrease in SBP ≥20 mmHg or in MAP ≥10 mmHg associated with a clinical event and need for nursing interventions. Patient’s self-assessment of QOL was evaluated by the 36-Item Short-Form Health Survey.

**Results:**

There were no significant associations between the mental summary score or the physical summary score and the proportion of dialysis sessions that fulfilled the full EBPG definition. A lower PRISS was significantly associated with the proportion of dialysis sessions that fulfilled the full EBPG definition (*R* = − 0.35, *P* = 0.0011), the proportion of dialysis sessions with a clinical event (*R* = − 0.64, *P* = 0.001), and the proportion of dialysis sessions with nursing interventions (*R* = − 0.41, *P* = 0.0001). The physical component summary and mental component summary were significantly negatively associated with the variable diabetes and positively with PRISS (*P* = 0.003 and *P* = 0.005, respectively). UF volume was significantly negatively associated with mental health (*P* = 0.02) and general health (*P* = 0.01).

**Conclusions:**

Our findings suggest that the EBPG definition of IDH does not capture aspects of intradialytic symptomatology that are relevant for the patient’s QOL. In contrast, we found a significant association between QOL and a simple patient-reported intra-dialytic symptom score, implying that how patients experience HD treatment influences their QOL.

**Electronic supplementary material:**

The online version of this article (10.1186/s12882-019-1366-2) contains supplementary material, which is available to authorized users.

## Background

In the past 10 to 15 years, there has been an increasing awareness that patient survival is not necessarily the main relevant outcome factor for patients with a chronic disease. Patient reported outcomes and Quality of Life (QOL) receive, with good reason, increasing attention in research regarding patients with chronic diseases, such as patients with end stage renal disease who depend on dialysis [1, 2]. To assess QOL, the RAND SF-36 (SF-36) has been proven to be beneficial for comparing general and specific populations, estimating the relative burden of different diseases, assessing the health benefits produced by a wide range of different treatments, and screening individual patients [3].

Intradialytic hypotension (IDH) is a serious and frequent complication of haemodialysis (HD) treatment [4, 5] It is often accompanied by symptoms such as nausea, dizziness, light-headedness, fatigue, and muscle cramps which affect the daily lives of HD patients [6] and, consequently, likely influence QOL. Pathophysiology of intradialytic hypotension and the methods to avoid this complication have been extensively investigated [7, 8]. Also, the association between IDH and mortality has been studied by several groups [7, 8], Flythe *et.al.* showed that an absolute nadir systolic blood pressure (SBP) < 90 mmHg was most potently associated with mortality [8]. In contrast, research on the association between intradialytic symptoms and QOL is minimal. Caplin *et.al.* studied the burden and duration of HD-associated symptoms with a survey but did not study the association between symptoms and QOL [6].

To support patients in effectively improving QOL, more knowledge is needed on the association between QOL and HD treatment-related factors like IDH. Furthermore, there is a need to identify aspects of IDH that have a (strong) effect on QOL. The goal of this study, therefore, was to determine whether the occurrence of IDH has an influence on the perception of QOL in HD patients. We studied this in a well-characterized patient group of 82 patients on maintenance HD over a period of 3 months comprising a total of 2623 HD-sessions. The focus of the study was on the association of QOL with the full definition of IDH according to the European Best Practice Guideline (EBPG) on haemodynamic instability as well as with its three components, i.e., a decrease in SBP of > 20 mmHg, the occurrence of clinical events, and nursing interventions [9]. To gain better insight into how the patients experienced the overall HD treatment, we additionally employed a simple patient-reported intradialytic symptom score (PRISS) that was filled out by the patients after each dialysis session.

## Subjects and methods

### Patients

This is a post-hoc analysis of a previous study on the prevalence of dialysis hypotension [10].

This multicenter prospective observational study included adult (≥18 years) patients from the Dialysis Center Groningen and the dialysis unit of the University Medical Center Groningen. Patients were eligible for the study when they satisfied the following criteria: maintenance bicarbonate HD for more than 3 months, three times per week, 3 ½ -4 ½ hours HD schedule. This study was approved by the Medical Ethical Committee of the University Medical Center Groningen. The Committee concluded that the Medical Research Involving Human Subjects Act (in Dutch: Wet Medisch-*wetenschappelijk Onderzoek met mensen)* was not applicable to this study (MEtc number: 2016/141). Obtaining oral informed consent was judged appropriate for this observational study that was conducted without intervention and without obtaining any patient material. All personal information was de-identified and analyzed anonymously. The study was performed in accordance with the principles of the Declaration of Helsinki.

### Study protocol

The design an methods of this study haven been previously reported [10]. In brief we prospectively collected the haemodynamic data, symptoms and nursing interventions of all of the HD sessions from participating patients during the 3 months of February, March, and April. All data were registered on a run sheet and stored electronically. The patients were asked to fill out a simple questionnaire after each HD session, i.e., a patient-reported intradialytic symptom score (PRISS). Patients scored how they had experienced the HD session on a 5 point Likert scale ranging from 0 (‘bad HD session’) to 5 (‘very good HD session’) [11]. Patient’s self-assessment of QOL was evaluated in the third month of the study by the 36-Item Short Form Health Survey (RAND SF-36) scoring system in the Dutch version [12]. The SF-36 consists of 36 questions in eight categories: physical functioning, physical role functioning, bodily pain, general health perceptions, vitality, social role functioning, emotional role functioning, and mental health. Among the eight categories, the four physical elements compose the physical component summary, and the emotional, mental and social functioning elements create the mental component summary.

Haemodialysis sessions during hospitalization were excluded from the analysis. Ultrafiltration rate was calculated by dividing ultrafiltration volume by dialysis session length and postdialysis body weight.

Cardiovascular history was defined as any history of heart disease, stroke, or peripheral vascular disease. Residual diuresis was defined as ≥200 ml/24 h. Equilibrated Kt/V was calculated from pre- and postdialysis plasma urea concentration according to the second-generation logarithmic Daugirdas equation [13].

Dialysis hypotension was defined according to the EBPG definition [9]: a decrease in SBP ≥20 mmHg or a decrease in MAP by ≥10 mmHg associated with a clinical event and need for nursing interventions. In additional analyses, we also used a decrease in SBP ≥30 and ≥ 40 mmHg as a designated limit. Patients were marked to have frequent dialysis hypotension when they fulfilled the EBPG definition of dialysis hypotension in ≥10% of dialysis sessions. The cut-off of 10% was arbitrarily chosen based on previous studies in which the prevalence of IDH ranged from 5 to 50% depending on the definition that was used [8, 10, 14–16]. Within this 5 to 50 range, we chose a relatively low cut-off of 10% since we used a strict definition of IDH.

### Statistical analysis

Data are reported as mean(±SD) for continuous variables with normal distributions, numbers (percent) for categorical data and median (interquartile range) for skewed data. The Shapiro Wilkinson test was used to test normality. Comparisons between groups with a normal distribution were made using a T-test, and for groups with a skewed distribution using the Mann Whitney U test. The Kruskal Wallis-test was used for multiple groups.

For the analysis of pre-, intra- and postdialysis haemodynamic parameters and PRISS, the data of all available HD sessions were averaged per patient. For the analysis of the components of QOL, a multivariate linear regression analysis with mixed effects model was utilized to identify QOL factors associated with IDH. This was followed by a model building strategy based on the Akaike Information Criterion (AIC model) [17–19]. Given the collection of possible models for the data, minimum AIC best selected the model by a maximum likelihood with a correction for overfitting. The following parameters were included in the model: age, gender, dialysis vintage, BMI, diabetic status, comorbid heart conditions, predialysis SBP, ultrafiltration volume, intradialytic clinical events, nursing interventions, PRISS and, various, a decrease in SBP of 20 mmHg, 30 mmHg, or 40 mmHg. Analyses were performed with SPSS version 20.0 (SPSS inc., IBM company, USA), GraphPad Prism version 5.0 and statistical programming language R version 3.4.0 (R Core Team, 2017).

## Results

### Patients

Of the 124 patients that participated in the original study, 82 patients filled out a QOL questionnaire. Patients who did not do so were not familiar with the Dutch language (*n* = 10), were mentally disabled (*n* = 4), or could not fill out a questionnaire due to intercurrent illness (*n* = 3). The reason for not filling out a questionnaire is unknown for 25 patients. There were no significant differences in characteristics between the patients who filled out the QOL questionnaire and those who did not.

The characteristics of the 82 patients are shown in Table 1. Mean (±SD) haemoglobin and albumin levels were 7.0 ± 0.8 mmol/l and 39.6 ± 3.1 g/l, respectively. eKt/V was 1.39 ± 0.26 per session. Haemodialysis access was an arteriovenous fistula or polytetrafluoroethylene (PTFE) graft in 82% of patients and a central venous catheter in 18% of patients. Cardiovascular medication was being used by 67% of the patients.

**Table 1:** Patient characteristics

Characteristic	*n* = 82
Age, year	64.1 ± 15.6
Dialysis vintage, months	32.0 ± 28.9
Males	41 (50%)
Diabetes	18 (22%)
Body mass index (kg/m^2^)	25.7 ± 5.0
Residual renal function	23 (28%)
Cardiovascular history	36 (44%)
Acute myocardial infarction	5 (6.1%)
Congestive heart failure	4 (4.9%)
Peripheral vascular disease	17 (20.7%)
Cerebral vascular disease	7 (8.5%)
Primary renal disease	
Hypertension	25 (31%)
Diabetes	11 (13%)
Glomerulonephritis	5 (6%)
Obstructive uropathy	14 (17%)
ADPKD	7 (9%)
IgA nephropathy	4 (5%)
Alports’ disease	1 (1%)
Other diagnoses	5 (6%)
Unknown	10 (12%)
Cardiovascular medication	
Beta-blocker	48 (59%)
CCB	21 (26%)
ACE-I/ ARB	16 (20%)

Note: continuous variables are presented as mean ± standard deviation

*Abbreviations*: *ADPKD* autosomal dominant polycystic kidney disease, *CCB* calcium channel blocker; *ACE-I* angiotensin converting enzyme inhibitor, *ARB* angiotensin receptor blocker

A total of 2623 HD sessions were analyzed with an average number of dialysis sessions per patient of 33 (range 14–36).

### Weight, ultrafiltration volume, blood pressure, and heart rate

The mean blood pressure decreased, from 145 ± 26 / 72 ± 15 mmHg predialysis to 130 ± 25 / 67 ± 14 mmHg at the end of the HD session. The mean MAP decreased from 96 ± 16 mmHg predialysis to 88 ± 17 mmHg postdialysis. The mean heart rate rose, from 75 ± 11 mmHg predialysis to 76 ± 14 bpm at the end of the HD sessions. The mean pre- and postdialysis body weight was 75.8 ± 15.4 kg and 73.9 ± 15.4 kg, respectively. The mean ultrafiltration volume and ultrafiltration rate in all 2623 dialysis sessions was 2457 ± 828 ml and 8.3 ± 3.1 ml/kg/hour, respectively.

IDH according to the full EBPG definition occurred in 6.7% of the HD sessions.

### Association of patient characteristics and intradialytic hypotension variables with QOL

For the QOL component physical functioning, younger patients had a significantly higher score (*P* = 0.003), and patients with a longer dialysis vintage had a considerably lower score (*P* = 0.002) (Additional file 1). Patients with diabetes scored notably higher on the QOL component pain (*P* = 0.04) (Additional file 1).

**Fig. 1:**
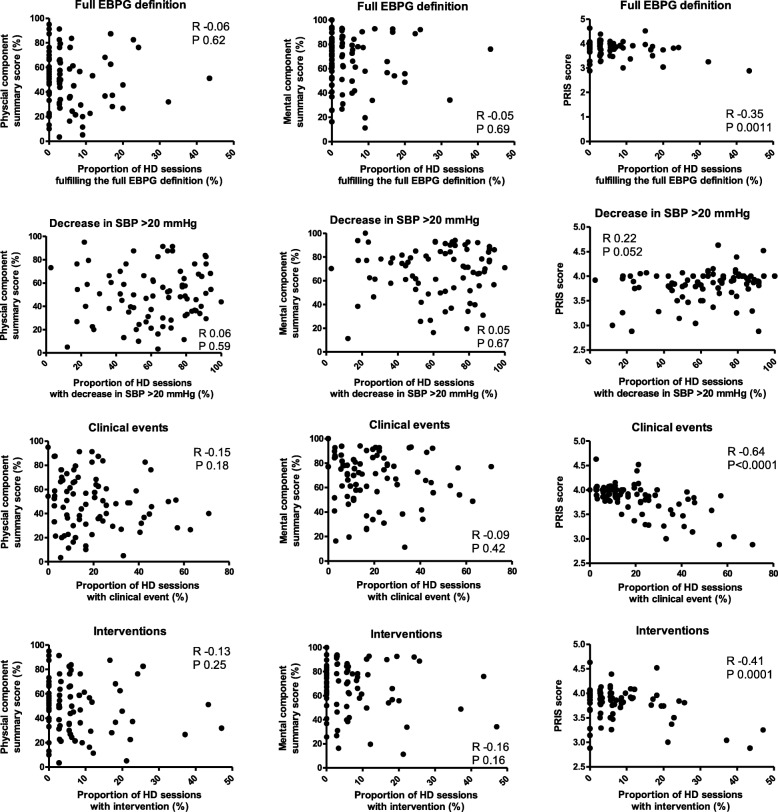
Scatterplots with correlations between the full EBPG definition (upper panel) and de EGBG components decrease in SBP ≥ 20 mmHg (second panel), clinical events (third panel) and nursing interventions (lowest panel) and physical component score, mental component score and PRISS

There were no significant associations between the mental summary score or the physical summary score and the proportion of dialysis sessions that fulfilled the full EBPG definition nor with the proportions of dialysis sessions that fulfilled one of the components of the EBPG definition (decrease in SBP of > 20 mmHg, clinical event, nursing interventions) (Fig. 1).

### Intradialytic hypotension variables and PRISS

There was no significant association between the PRISS and the proportion of dialysis sessions in which a decrease in SBP of > 20 mmHg occurred. A lower PRISS was significantly associated with the proportion of dialysis sessions that fulfilled the full EBPG definition (*R* = − 0.35, *P* = 0.0011), the proportion of dialysis sessions with a clinical event (*R* = − 0.64, *P* = 0.001), and the proportion of dialysis sessions with nursing interventions (*R* = − 0.41, *P* = 0.0001) (Fig. 1).

A lower PRISS score was significantly associated with a lower score for the QOL components general health (*P* = 0.02), health change (*P* = 0.03), and the physical summary score (*P* = 0.02).

### Multivariable analyses

In the multivariate linear regression model with optimizing AIC, the outcome variables of physical component summary and mental component summary were significantly negatively associated with the variable diabetes and positively with PRISS (*P* = 0.003 and *P* = 0.005, respectively) (Table 2). The response variable physical functioning was significantly negatively associated with age (*P* = 0.00), dialysis vintage (*P* = 0.04) and PRISS (*P* = 0.004), and also negatively but not significantly with BMI (*P* = 0.10). BMI was significantly negatively associated with the response variable social functioning (*P* = 0.05). The response variable emotional role functioning was negatively but not significantly associated with nursing interventions (*P* = 0.08) and total UF volume (*P* = 0.15) and significantly positively associated with clinical events (*P* = 0.005) and with PRISS (*P* = 0.01) (Additional file 2). UF volume was also negatively but not significantly associated with QOL components social functioning (*P* = 0.16), physical role functioning (*P* = 0.14), and emotional role functioning (*P* = 0.15) and significantly negatively associated with mental health (*P* = 0.02) and general health (*P* = 0.01). These analyses included a decrease in SBP of ≥20 mmHg as a correcting explanatory variable. Analyses with predialysis SBP, a decrease in SBP of ≥30 mmHg and ≥ 40 mmHg showed identical results.

**Table 2:** Multivariate linear regression analysis with model building strategy Akaike Information Criterion (AIC); factors associated with Quality of life Summary scores

		Gender	Age	Dialysis vintage	BMI	Diabetes	CV comorbidity	Decrease in SBP > 20 mmHg (%)	Clinical events (%)	Interventions (%)	Total UF	PRISS	Adjusted R
Physical Component summary	Estimate					−12.00						23.02	
	95% CI					−23.69 to-0.31						7.89 to 38.14	
	SE					5.87						7.60	
	P					0.04^a^						0.003^a^	0.10
Mental component summary	Estimate					−9.36			0.30		0.01	27.91	
	95% CI					−20.88 to 2.16			−0.09 to 0.68		−0.01 to 0.00	8.80 to 47.03	
	SE					5.78			0.19		0.003	9.59	
	P					0.11			0.13		0.06	0.005^a^	0.10

*Est* Estimate, *SE* Standard Error, *T* T-value, *P P*-value, *CI* Confidence Interval, *U* Upper bound, *L* Lower bound, ^a^ significant, *UF* Ultrafiltration volume, *CV* Cardiovascular, *R* the variance of the QOL variables explained by the explanatory variables (in %)

## Discussion

In this study we found that there is no association between QOL and IDH as defined according to the EBPG guideline. This is factual for the standard EBPG definition as well as when a decrease in SBP of ≥30 or ≥ 40 mmHg is chosen as the blood pressure decline component instead of a decrease in SBP ≥20 mmHg or a decrease in MAP ≥10 mmHg. These findings suggest that the EBPG definition of IDH does not capture aspects of intradialytic symptomatology that are relevant for QOL. In contrast, we found a significant association between QOL and a simple patient-reported intradialytic symptom score, i.e., the PRISS, indicating that the way patients experience HD treatment indeed influences QOL.

The association between age and dialysis vintage with the physical functioning component of the QOL was expected and is explained by deteriorating physical function as patients become older and are on the HD treatment for a longer period of time [10, 20]. BMI was significantly negatively associated with social functioning. Although HD patients with a higher BMI have been reported to have better survival, a higher BMI may be associated with a lower QOL in this population [21]. The association between diabetes and the QOL components, emotional functioning, and pain are also in accordance with previous studies and are explained by a higher prevalence of cardiovascular complication and diabetic complications such as neuropathy [22, 23].

Our analyses show the association between QOL and UF-volume, clinical events, and nursing interventions which are parameters that are directly or indirectly related to fluid restriction. For some patients, this is very difficult to maintain, and this may cause stress and anxiety.

The analysis of IDH is complicated by the wide variation in definitions of dialysis hypotension that are used in the literature [10]. Some definitions only use a minimum decrease (e.g., ≥20 or ≥ 30 mmHg) in SBP [24–26]. Other definitions require a combination of symptoms and interventions with a fall in blood pressure [9, 16, 27]. In this study, there was no association encountered between a decrease in SBP of either ≥20, ≥30, or ≥ 40 mmHg and QOL. This finding suggests that a reduction in SBP does not have a major impact on QOL in HD patients. In our previous article we described that various factors may affect predialysis blood pressure, the transportation to the dialysis unit can be stressfull as well as puncturing the fistula. In this regard the predialysis blood pressure may not be a valid value as reference point, as the early intradialytic fall in blood pressure may be explained by the relief of stress/anxiety, and not by dialysis-specific haemodynamic stress [10].

Presently, there is no general consensus regarding the best evidence-based indicators of IDH. We agree with, e.g., Assismon et al., that the lack of such indicators has hindered the data synthesis and the development of evidence-based guidelines for the prevention and treatment of IDH as well as prevented an accurate estimation of the population burden of IDH and patient risk assessment [28].

An absolute nadir intradialytic BP of SPB < 90 mmHg was previously found to be associated with an increased mortality risk; however, intradialytic symptoms and interventions were not associated with this risk [8]. An important question is whether mortality can be lowered by preventing a decrease in SBP to < 90 mmHg. This may depend on the type of preventive measures that are taken. Increasing dry weight or preventive intradialytic administration of saline carries the risk of chronic overhydration which has a strong negative impact on survival [29].

Further research is needed to understand the underlying mechanisms of the IDH related symptoms and to provide the patient with the optimal dialysis treatment [30, 31]. The finding that the way patients experience HD treatment influences QOL may underscore the impact of dialysis on their personal life, not only for the patient but, most likely, also for their family members [32]. This information can be used by medical and nursing staff to provide a frame of reference to better understand the consequences on the daily life of patients. In addition to focusing on the medical condition and the blood pressure course during the HD treatment, more attention to the factors that influence QOL seems beneficial for patients.

An important observation is that clinical symptoms and nursing interventions are not hard endpoints and subject to bias, with variation between patients in reporting symptoms and the threshold to start an intervention between healthcare professionals. This also applies for how patients interpreted their QOL and symptoms and rated the HD treatment in the PRISS. The PRISS is a 5-point Likert scale measuring a positive or negative response to a statement which was suitable for the question of how they had experienced the HD session. The validity of the Likert Scale attitude measurement can be compromised due to social desirability. The SF-36 does not include symptoms and problems that are specific to a particular condition, but SF-36 scales correlate substantially with most of the omitted general health concepts and with the frequency and severity of many specific symptoms [33]. Relative to other published measures, the mental health, role- emotional, and social functioning scale and the mental component summary have been shown to be the most valid mental health measures in the method of known groups-validity. The physical functioning, role- physical, and bodily pain scales and the physical component summary have shown to be the most valid physical health measures [33]. Future studies should preferably use the QDQOL, since this tool is supplemented with multi-item scales targeted at particular concerns of individuals with a kidney disease and on dialysis. The number of patients in our study is relatively low. However, the long study duration of 3 months as well as the frequent measurement of blood pressure and the post-dialytic recording of the PRISS (and active search for patient complaints at each dialysis session) reduced the possibility of underestimation of dialysis hypotension. Another limitation of our study is that we did not take into account seasonal variations in BP. Our study was performed in February through April and, therefore we do not have information on seasonal variations. We also acknowledge that the results in our Dutch cohort may not be representative for other populations that have a higher incidence of diabetes and overweight and higher ultrafiltration rates.

## Conclusion

Our findings suggest that the EBPG definition of IDH does not capture aspects of intradialytic symptomatology that are relevant for the patient’s QOL. In contrast, we found a significant association between QOL and a simple patient-reported intra-dialytic symptom score, i.e., the PRISS, indicating that how patients experience HD treatment influences their QOL. Further research is needed to confirm our findings and to refine the definition of IDH based on the purpose for which the definition is used. More attention to the impact of symptom burden of HD treatment is helpful for improving the QOL of HD patients.

## Additional files

Additional file 1: Patient characteristics associated with Quality of life components. (DOCX 18 kb)

Additional file 2: Multivariate lineair regression analysis with model building strategy Akaike Information Criterion (AIC); factors associated with Quality of life components. (DOCX 19 kb)

## Abbreviations

BIC: Bayesian Information Criterion
EBPG: European Best Practice Guideline
HD: Haemodialysis
IDH: Intradialytic hypotension
MAP: Mean arterial pressure
PRISS: Patient reported outcome symptom score
PTFE: Polytetrafluoroethylene
QOL: Quality of Life
SBP: Systolic blood pressure
SF-36: 36-Item Short Form Health Survey
UF: Ultrafiltration
