# Supplementary material for: Association between quality of life and various aspects of intradialytic hypotension including patient-reported intradialytic symptom score
Source: BMC Nephrol. 2019 May 14;20:164. doi: 10.1186/s12882-019-1366-2 (PMC6518736; doi:10.1186/s12882-019-1366-2)
Supplement: Supplementary file 1 — Patient characteristics associated with Quality of life components. (DOCX 18 kb) [file 12882_2019_1366_MOESM1_ESM.docx]

**Additional file 1.**

**Patient characteristics associated with Quality of life components.**

|  | **Gender** | | **Age** | | **Dialysis vintage** | | | **Diabetes** | | **CV comorbidity** | |
| --- | --- | --- | --- | --- | --- | --- | --- | --- | --- | --- | --- |
|  | M | F | ≤50 | >50 | ≤12 | >12-≤36 | >36 | Y | N | Y | N |
| **N** | 41 | 41 | 16 | 66 | 20 | 37 | 25 | 18 | 64 | 36 | 46 |
| **Physical functioning**  **P** | 52.5  (31.3-75.0)  0.45 | 45  (25.0-75.0) | 77.5  (52.5-85.0)  0.003* | 40.0  (25.0-65.0) | 47.5  (27.5-75.0) | 60.0  (36.3-83.8)  0.02* | 35.0  (17.5-50.0) | 37.5  (23.8-76.3)  0.39 | 50  (30.0-75.0) | 45.0  (31.3-68.8)  0.82 | 50.0  (25.0-80.0) |
| **Social functioning**  **P** | **75.0**  **(62.5-93.8)**  **0.71** | **75.0**  **(56.3-87.5)** | **68.8**  **(53.1-84.4)**  **0.12** | **87.5**  **(62.5-100)** | **68.8**  **(40.6-87.5)** | **87.5**  **(68.8-100)**  **0.07** | **75.0**  **(56.3-87.5)** | **75.0**  **(50.0-87.5)**  **0.46** | **75.0**  **(62.5-96.9)** | **87.5**  **(75.0-100)**  **0.02*** | **75.0**  **(50.0-87.5)** |
| **Physical role functioning**  **P** | **25.0**  **(00.0-100)**  **0.07** | **00.0**  **(00.0-62.5)** | **00.0**  **(00.0-62.5)**  **0.32** | **25.0**  **(00.0-100)** | **25.0**  **(00.0-68.8)** | **25.0**  **(00.0-100)**  **0.22** | **00.0**  **(00.0-37.5)** | **00.0**  **(00.0-62.5)**  **0.52** | **25.0**  **(00.0-75.0)** | **25.0**  **(00.0-100)**  **0.25** | **00.0**  **(00.0-75.0)** |
| **Emotional role functioning**  **P** | 100  (33.3-100)  0.78 | 100  (33.3-100) | 100  (33.3-100)  0.64 | 100  (33.3-100) | 100  (58.3-100) | 100  (58.3-100)  0.01* | 33.3  (00.0-100) | 66.7  (00.0-100)  0.13 | 100  (33.3-100) | 100  (33.3-100)  0.55 | 100  (00.0-100) |
| **Mental health**  **P** | **80.0**  **(60.0-96.0)**  **0.59** | **80.0**  **(64.0-88.0)** | **68.0**  **(52.0-88.0)**  **0.11** | **82.0**  **(64.0-92.0)** | **76.0**  **(52.0-88.0)** | **80.0**  **(64.0-95.0)**  **0.56** | **80.0**  **(64.0-92.0)** | **80.0**  **(54.0-90.0)**  **0.48** | **80.0**  **(64.0-92.0)** | **84.0**  **(66.0-96.0)**  **0.17** | **76.0**  **(64.0-88.0)** |
| **Vitality**  **P** | **55.0**  **(40.0-70.0)**  **0.20** | **45.0**  **(30.0-63.8)** | **45.0**  **(31.3-58.8)**  **0.08** | **55.0**  **(35.0-70.0)** | **45.0**  **(35.0-60.0)** | **55.0**  **(37.5-70.0)**  **0.51** | **55.0**  **(30.0-70.0)** | **50.0**  **(27.5-75.0)**  **0.98** | **55.0**  **(35.0-70.0)** | **50.0**  **(40.0-70.0)**  **0.91** | **55.0**  **(32.5-70.0)** |
| **Bodily Pain**  **P** | 87.8  (51.0-100)  0.37 | 67.3  (44.9-100) | 67.3  (53.5-94.9)  0.59 | 87.8  (44.9-100) | 67.3  (44.9-100) | 89.8  (62.2-100)  0.14 | 67.3  (39.8-100) | 44.9  (34.2-100)  0.04* | 83.7  (55.1-100) | 83.7  (44.9-100)  0.66 | 78.6  (44.9-100) |
| **General Health**  **P** | 45  (25.0-56.3)  0.75 | 42.5  (25.0-58.8) | 37.5  (16.3-50.0)  0.13 | 45.0  (28.8-60.0) | 40.0  (25.0-60.0) | 45.0  (35.0-62.5)  0.39 | 42.5  (18.8-55.0) | 42.5  (20.0-55.0)  0.63 | 45.0  (25.0-60.0) | 40.0  (25.0-56.3)  0.52 | 45.0  (25.0-58.8) |
| **Health change**  **P** | 50  (50.0-75.0)  0.09 | 50  (25.0-62.5) | 50.0  (25.0-75.0)  0.30 | 50.0  (25.0-75.0) | 50.0  (31.3-75.0) | 50.0  (25.0-75.0)  0.25 | 50.0  (25.0-50.0) | 75.0  (43.8-81.3)  0.12 | 50.0  (25.0-75.0) | 50.0  (25.0-75.0)  0.68 | 50.0  (43.8-75.0) |
| **Physical Component Summary**  **P** | 51.8  (36.8-69.4)  0.22 | 46.3  (27.4-63.4) | 50.9  (35.2-58.4)  0.82 | 48.1  (29.6-66.9) | 51.2  (25.8-65.6) | 56.5  (37.0-74.7)  0.03* | 39.9  (28.1-50.6) | 34.9  (23.6-60.0)  0.12 | 50.6  (36.2-66.3) | 47.8  (34.1-65.6)  0.89 | 49.9  (31.5-67.2) |
| **Mental Component Summary**  **P** | 72.3  (52.8-86.1)  0.58 | 68  (54.5-80.4) | 70.9  (46.8-76.8)  0.22 | 72.3  (54.1-86.4) | 71.3  (51.0-77.0) | 78.3  (61.1-87.6)  0.16 | 67.5  (39.8-79.1**)** | 65.8  (45.7-81.4)  0.46 | 72.5  (57.6-84.4) | 76.4  (56.9-87.1)  0.20 | 67.0  (52.2-81.1) |

**Continue variables are presented as Median (IQR), Abbreviations: * =significant, CV = Cardiovascular**
