# Supplementary material for: Association between quality of life and various aspects of intradialytic hypotension including patient-reported intradialytic symptom score
Source: BMC Nephrol. 2019 May 14;20:164. doi: 10.1186/s12882-019-1366-2 (PMC6518736; doi:10.1186/s12882-019-1366-2)
Supplement: Supplementary file 2 — Multivariate lineair regression analysis with model building strategy Akaike Information Criterion (AIC); factors associated with Quality of life components. (DOCX 19 kb) [file 12882_2019_1366_MOESM2_ESM.docx]

**Additional file 2.**

**Multivariate linear regression analysis with model building strategy Akaike Information Criterion (AIC); factors associated with Quality of life components.**

|  |  | **Gender** | **Age** | **Dialysis vintage** | **BMI** | **Diabetes** | **CV comorbidity** | **Decrease in SBP >20 mmHg (%)** | **Clinical events (%)** | **Interventions (%)** | **Total UF** | **PRISS** | **Adjusted R** |
| --- | --- | --- | --- | --- | --- | --- | --- | --- | --- | --- | --- | --- | --- |
| **Physical functioning** | Estimate |  | -0.80 | -0.21 | -0.93 |  |  |  |  |  |  | 26.08 |  |
|  | 95% CI |  | -1.16 to -0.45 | -0.41 to 0.01 | -2.04 to 0.18 |  |  |  |  |  |  | 8.28 to 43.87 |  |
|  | SE |  | 0.18 | 0.09 | 0.56 |  |  |  |  |  |  | 8.93 |  |
|  | P |  | 0.00** | 0.04* | 0.10 |  |  |  |  |  |  | 0.004* | 0.28 |
| **Social functioning** | Estimate |  |  |  | -1.11 |  | 8.91 |  |  |  | -0.005 | 15.58 |  |
|  | 95% CI |  |  |  | -2.21 to -0.01 |  | -1.88 to 19.70 |  |  |  | -0.01 to 0.002 | -1.43 to 32.59 |  |
|  | SE |  |  |  | 0.55 |  | 5.42 |  |  |  | 0.003 | 8.54 |  |
|  | P |  |  |  | 0.05* |  | 0.10 |  |  |  | 0.17 | 0.07 | 0.085 |
| **Physical role functioning** | Estimate | -16.94 |  |  |  |  |  | 0.28 |  |  | -0.01 |  |  |
|  | 95% CI | -35.76 to 1.87 |  |  |  |  |  | -0.12 to 0.69 |  |  | -0.02 to 0.003 |  |  |
|  | SE | 9.44 |  |  |  |  |  | 0.20 |  |  | 0.01 |  |  |
|  | P | 0.08 |  |  |  |  |  | 0.17 |  |  | 0.14 |  | 0.030 |
| **Emotional role functioning** | Estimate |  |  | -0.24 |  | -22.51 |  |  | 1.18 | -0.99 | -0.01 | 49.41 |  |
|  | 95% CI |  |  | -0.56 to 0.08 |  | -44.72 to -0.31 |  |  | 0.37 to 1.99 | -2.09 to 0.11 | -0.02 to 0.003 | 12.14 to 86.67 |  |
|  | SE |  |  | 0.16 |  | 11.13 |  |  | 0.41 | 0.55 | 0.01 | 18.68 |  |
|  | P |  |  | 0.14 |  | 0.05* |  |  | 0.005* | 0.08 | 0.15 | 0.01* | 0.16 |
| **Mental health** | Estimate |  |  |  |  |  |  |  |  |  | -0.01 |  |  |
|  | 95% CI |  |  |  |  |  |  |  |  |  | -0.012 to-0.001 |  |  |
|  | SE |  |  |  |  |  |  |  |  |  | 0.003 |  |  |
|  | P |  |  |  |  |  |  |  |  |  | 0.02* |  | 0.053 |
| **Vitality** | Estimate |  | 0.25 |  |  |  |  |  |  |  |  | 13.57 |  |
|  | 95% CI |  | -0.06 to 0.55 |  |  |  |  |  |  |  |  | -1.04 to 28.18 |  |
|  | SE |  | 0.15 |  |  |  |  |  |  |  |  | 7.34 |  |
|  | P |  | 0.11 |  |  |  |  |  |  |  |  | 0.07 | 0.075 |
| **Bodily Pain** | Estimate |  |  |  |  | -20.78 |  |  |  |  |  | 27.13 |  |
|  | 95% CI |  |  |  |  | -35.68 to -5.88 |  |  |  |  |  | 7.86 to 46.41 |  |
|  | SE |  |  |  |  | 7.49 |  |  |  |  |  | 9.68 |  |
|  | P |  |  |  |  | 0.007* |  |  |  |  |  | 0.006* | 0.12 |
| **General Health** | Estimate |  |  |  |  |  |  |  | 0.27 |  | -0.01 | 30.13 |  |
|  | 95% CI |  |  |  |  |  |  |  | -0.10 to 0.65 |  | -0.01 to-0.002 | 12.28 to 47.99 |  |
|  | SE |  |  |  |  |  |  |  | 0.19 |  | 0.003 | 8.95 |  |
|  | P |  |  |  |  |  |  |  | 0.15 |  | 0.01* | 0.001* | 0.17 |
| **Health change** | Estimate |  |  |  |  |  |  |  |  |  |  | 34.55 |  |
|  | 95% CI |  |  |  |  |  |  |  |  |  |  | 16.88 to 52.23 |  |
|  | SE |  |  |  |  |  |  |  |  |  |  | 8.88 |  |
|  | P |  |  |  |  |  |  |  |  |  |  | 0.00* | 0.15 |

**Es t= Estimate, SE= Standard Error, P= P-value, CI= Confidence Interval, U =Upper bound,L= Lower bound, * =significant, UF =Ultrafiltration volume, CV = Cardiovascular, R =the variance of the QOL variables explained by the explanatory variables (in %).**
